# Microplotter Printing of a Miniature Flexible Supercapacitor Electrode Based on Hierarchically Organized NiCo_2_O_4_ Nanostructures

**DOI:** 10.3390/ma16124202

**Published:** 2023-06-06

**Authors:** Tatiana L. Simonenko, Nikolay P. Simonenko, Philipp Yu. Gorobtsov, Elizaveta P. Simonenko, Nikolay T. Kuznetsov

**Affiliations:** Kurnakov Institute of General and Inorganic Chemistry of the Russian Academy of Sciences, 31 Leninsky Pr., Moscow 119991, Russia; egorova.offver@gmail.com (T.L.S.); phigoros@gmail.com (P.Y.G.); ep_simonenko@mail.ru (E.P.S.); ntkuz@igic.ras.ru (N.T.K.)

**Keywords:** hydrothermal synthesis, hierarchical organization, NiCo_2_O_4_, spinel, microplotter printing, electrode, planar supercapacitor, flexible electronics

## Abstract

The hydrothermal synthesis of a nanosized NiCo_2_O_4_ oxide with several levels of hierarchical self-organization was studied. Using X-ray diffraction analysis (XRD) and Fourier-transform infrared (FTIR) spectroscopy, it was determined that under the selected synthesis conditions, a nickel-cobalt carbonate hydroxide hydrate of the composition M(CO_3_)_0.5_(OH)·0.11H_2_O (where M–Ni^2+^ and Co^2+^) is formed as a semi-product. The conditions of semi-product transformation into the target oxide were determined by simultaneous thermal analysis. It was found by means of scanning electron microscopy (SEM) that the main powder fraction consists of hierarchically organized microspheres of 3–10 μm in diameter, and individual nanorods are observed as the second fraction of the powder. Nanorod microstructure was further studied by transmission electron microscopy (TEM). A hierarchically organized NiCo_2_O_4_ film was printed on the surface of a flexible carbon paper (CP) using an optimized microplotter printing technique and functional inks based on the obtained oxide powder. It was shown by XRD, TEM, and atomic force microscopy (AFM) that the crystalline structure and microstructural features of the oxide particles are preserved when deposited on the surface of the flexible substrate. It was found that the obtained electrode sample is characterized by a specific capacitance value of 420 F/g at a current density of 1 A/g, and the capacitance loss during 2000 charge–discharge cycles at 10 A/g is 10%, which indicates a high material stability. It was established that the proposed synthesis and printing technology enables the efficient automated formation of corresponding miniature electrode nanostructures as promising components for flexible planar supercapacitors.

## 1. Introduction

The development of flexible, compact, portable microelectronic devices, including wearable ones, requires the creation of efficient, flexible electric energy storage systems. To date, the most promising from this point of view are lithium-ion batteries (LIBs) and supercapacitors [1,2,3,4]. However, it is necessary to consider that accumulators are usually characterized by a smaller working cycle quantity in comparison with supercapacitors. In addition, components used in LIBs are frequently unstable in the air and toxic, which can impose strict requirements on their sealing and compliance with certain rules of storage, operation, and utilization of such devices. On the other hand, supercapacitors demonstrate higher power density values than batteries, an increased charge–discharge rate, and can be designed from relatively safe materials, which makes these devices easier to handle and more environmentally friendly [5,6]. The substrate material selection is one of the key tasks in the formation of flexible planar-type energy storage devices. Such parameters as flexibility, thickness, and roughness of the substrate, as well as its density, thermal stability, and wettability, are important in this case [7,8,9]. Recently, carbon materials, in particular, carbon paper, which is characterized by high electrical conductivity, has adequate strength, toughness, and flexibility, and is commercially available, have attracted considerable interest in this regard [10,11,12].

Conducting polymers (polyaniline, polythiophene, poly-3,4-ethylenedioxythiophene:polystyrene sulfonate, polypyrrole, etc.), individual hydroxides, and transition metal oxides, as well as complex oxides that exhibit pseudocapacitative properties are most commonly used as the active material for supercapacitor electrodes [5,13]. Polymer-based electrodes are usually characterized by high conductivity values and can be used as both electrodes and current collectors in planar supercapacitors without the need for extra conductive additives. Nevertheless, the cyclic processes of significant expansion and contraction of polymeric electrodes during their operation have a negative impact on the corresponding devices’ stability [14]. At the same time, transition metal oxides of complex composition, in particular spinels having the general formula AB_2_O_4_, demonstrate higher specific capacity values than those of polymers and are characterized by high electrochemical activity due to the presence of two metal cations with variable oxidation degree in their composition [15,16,17]. One of the most in demand in this context oxides with a spinel-type structure is NiCo_2_O_4_ [18,19,20]. This material’s advantages include the high specific capacitance values of supercapacitors based on it (can reach 2876 F/g [21]) and a long operating life [22,23], as well as commercial availability and low toxicity [24].

It is known that the functional characteristics of materials are determined not only by their chemical composition and dispersity but also by their microstructure. A number of papers [25,26,27,28,29,30] have demonstrated that obtaining hierarchically organized nanostructures, including those consisting of anisotropic particles, when creating materials for modern energy storage devices allows the length of the charge (ion/electron) transfer path to be reduced, the active surface area to be increased, and the bulk density of the resulting electrode materials to be decreased. This, in turn, contributes to increasing electrochemical activity and capacitance of electrodes, improving their microstructural stability during cyclic charging–discharging. According to the literature, one of the most promising and popular methods for obtaining hierarchically organized oxide nanostructures is hydrothermal synthesis, which enables the formation of the widest range of different morphologies: hollow spheres [31], nanotubes, nanofibers and nanorods [25,32,33,34], nanosheets and plate-like structures [35,36,37], or nanoflowers [26,38,39], both as nanopowders and as coatings. At the same time, a number of papers [40,41] note the high potential of the NiCo_2_O_4_ microstructure, consisting of nanorods organized in the form of spheres (“urchin-like” microstructure), with respect to improving the electrochemical characteristics of their supercapacitor electrodes (high specific capacitance values, low charge transfer resistance, and increased cyclic stability).

The formation of electrode nanostructures of a given geometry on different substrates requires the involvement of modern high-tech methods and approaches. Recently, the trend towards additive technologies (3D printing [42,43], inkjet printing [44,45,46], pen plotter printing [47,48], microplotter printing [49] and microextrusion printing [50], roll-to-roll printing [51,52], transfer printing [53,54], and screen printing [55,56]) has been increasingly evident in the formation of various electronic and alternative energy devices, including supercapacitors. The above approaches enable the automated formation of different (including complex) geometries with a high degree of reproducibility, precise dosage, and addressability of material application, along with an opportunity to efficiently scale up the process while maintaining the microstructural and functional characteristics of the manufactured devices. From this list of printing technologies, it is necessary to highlight microplotter printing as one of the new, insufficiently studied varieties of additive technologies in terms of inorganic coating formation. Application of this printing method in our work made it possible to form coatings of the desired composition while preserving the target coating nanoarchitecture inherited from the nanopowder used for the functional ink preparation. In addition, we have shown that microplotter printing is a convenient approach for applying coatings to the flexible substrate surface (in particular, carbon paper), which opens up broad prospects for this printing approach in the planar supercapacitor design for the flexible and wearable electronics industry.

Thus, this work was aimed at studying the urchin-like NiCo_2_O_4_ hydrothermal synthesis and developing a technique for the microplotter printing of a hierarchically organized electrode film based on it on a flexible carbon paper substrate. In addition, the prospects of the proposed synthesis and printing approach for creating flexible supercapacitors components were demonstrated. 

## 2. Materials and Methods

### 2.1. NiCo_2_O_4_ Nanopowder Obtaining

NiCo_2_O_4_ oxide nanopowder was prepared by the hydrothermal method. Therefore, urea ((NH_2_)_2_CO, 99%, RUSHIM, Moscow, Russia) was added (c = 0.3 mol/L) to an aqueous solution of nickel chloride (NiCl_2_·6H_2_O, >98%, Lenreactiv, St. Petersburg, Russia) and cobalt nitrate (Co(NO_3_)_2_·6H_2_O, >98%, Lenreactiv, St. Petersburg, Russia); the total metals concentration was 0.1 mol/L, n(Co)/n(Ni) = 2. After all the components dissolved, the reaction system was placed in a Teflon-lined autoclave and underwent a programmed heat treatment in a muffle furnace (temperature—180 °C, heat treatment duration—1 h, heating rate—1.5 °/min), followed by natural cooling. The solid phase formed as a result of the synthesis was separated from the mother liquor and washed with distilled water through cyclic centrifugation. Then, the resulting precipitate was dried at 100 °C (6 h) with subsequent additional heat treatment at 400 °C (5 h) for crystallization of the target oxide.

### 2.2. Microplotter Printing of Active Electrode Layer

**Figure 1:**
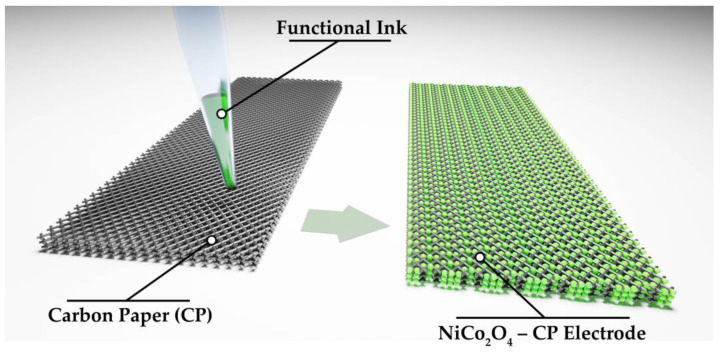
Illustration of the NiCo_2_O_4_ film microplotter printing process on the CP surface.

During the next stage, a stable dispersed system (solid phase particle content of 5 wt%) suitable in its rheological characteristics for application as a functional ink for microplotter printing (Figure 1) of NiCo_2_O_4_ coating on carbon paper substrate (substrate dimensions 21 × 8 × 0.1 mm) was obtained with the formed oxide nanopowder and ethanol (gradient HPLC grade, Chimmed, Moscow, Russia). The printing process was preceded by the digital pathway design, according to which the discrete dispensing of individual ink microdoses onto the substrate surface was performed with the help of a 3-axis positioning system, alternating with the automated dispenser (100 μm in diameter) filling. The optimum displacement speed of the dispenser was 100 mm/min. Thus, a coating was obtained on the carbon paper (lateral dimensions were 18 × 8 mm), which then underwent drying at 35 °C (2h) to remove the solvent. The total mass loading was about 0.5 mg/cm^2^.

### 2.3. Instrumentation

The thermal behavior of the semi-product obtained from hydrothermal synthesis and subsequent drying was studied with the use of a TGA/DSC thermal analyzer (SDT Q-600, TA Instruments, New Castle, DE, USA) in the 25–1000 °C range in the following mode: the heating rate was 10 °/min, airflow was 250 mL/min; and the analyzed sample mass was 7.128 mg.

Functional groups contained in the studied powders were detected on an InfraLUM FT-08 FTIR spectrometer (Lumex, St. Petersburg, Russia). Transmission spectra of suspensions on the basis of obtained powders in Vaseline oil, placed as films between glasses of potassium bromide, were recorded in the 350–4000 cm^−1^ interval (signal accumulation time—15 s, resolution—1 cm^−1^).

X-ray diffraction analysis of the samples was performed with a D8 Advance diffractometer (Bruker, Bremen, Germany; CuKα = 1.5418 Å, Ni-filter, E = 40 keV, I = 40 mA, 2θ range—5–80°, resolution—0.02°, the signal accumulation time in a single point was 0.3 s and 2.0 s in the 2θ range—28–45°). XRD pattern analysis was carried out using the Rietveld refinement method, implemented in X’Pert HighScore Plus software, version 3.0e (PANalytical B.V., Almelo, The Netherlands).

The microstructural properties of the obtained nanopowders and the film printed on the CP surface were examined by scanning electron microscopy (NVision-40, Carl Zeiss, Inc., Oberkochen, Germany) at an accelerating voltage of 1 kV. Elemental analysis and elemental mapping of the film surface were performed by SEM using an EDX spectrometer INCA X-MAX 80 (Oxford Instruments, Abingdon, UK) at an accelerating voltage of 20 kV and a focal distance of 5 mm. Additional analysis of the obtained powder morphology and selected area electron diffraction (SAED) was performed by TEM and high-resolution TEM (HR-TEM; JEOL JEM-1011 with ORIUS SC1000W digital camera; JEOL JEM2100; JEOL Ltd., Akishima, Tokyo, Japan).

Atomic force microscopy was also used to study the film printed on the CP substrate surface. Data on the film microstructure and its local electrophysical properties were obtained as a result of the measurements. For this purpose, a Solver Pro-M scanning probe microscope (NT-MDT, Zelenograd, Russia) and ETALON HA-HR probes (ScanSens, Bremen, Germany) with W_2_C+ conductive coating (<35 nm radius) in the modes of semicontact AFM and Kelvin-probe scanning microscopy (KPFM) were used. Measurements were carried out at atmospheric pressure.

Electrochemical characterization of the electrode sample was performed on a P-40X potentiostat-galvanostat equipped with an electrochemical impedance measurement module FRA-24M (Electrochemical Instruments, Chernogolovka, Russia). The investigated material printed on the carbon paper served as a working electrode, and Ag/AgCl and graphite rods were used as reference and counter electrodes, respectively. Cyclic voltammetry (CVA), galvanostatic charge–discharge (GCD), and electrochemical impedance spectroscopy (EIS) measurements were carried out in the electrolyte environment, which was an aqueous solution of KOH (c = 3 mol/L). The specific capacity (C_g_) of the electrode material under study was calculated using the discharge data recorded in the three-electrode scheme according to the following formula:

C_g_ = (I × Δt)/m × ΔV, (1)

where I is the value of direct current applied (A), Δt is the discharge time (s), m is the mass of the active electrode material (g), and ΔV is the discharge potential window (V).

## 3. Results and Discussion

### 3.1. Investigation of the Prepared Nanopowder

**Figure 2:**
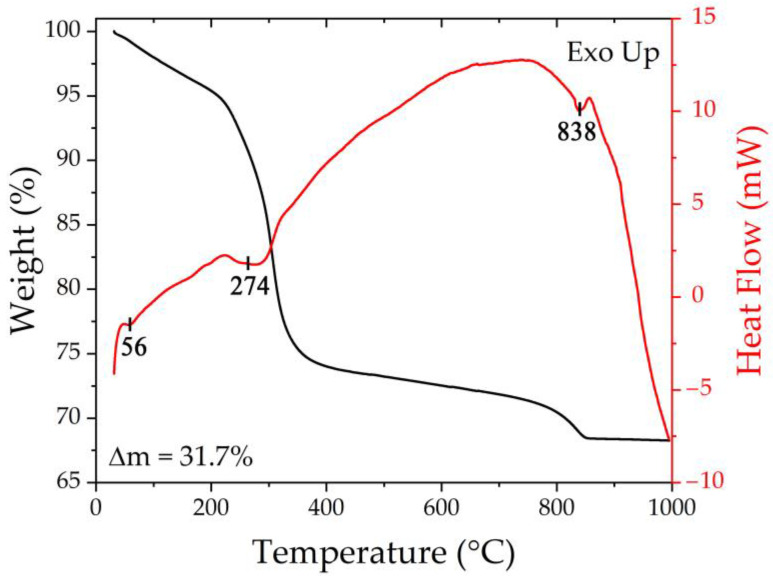
Thermal analysis results for the resulting semi-product.

Synchronous thermal analysis was used to determine the optimal conditions for additional heat treatment of the obtained semi-product powder in order to crystallize the target NiCo_2_O_4_ oxide. According to the thermograms (Figure 2), the powder heating in the range of 25–1000 °C resulted in 3-step weight loss in the range of 25–250, 250–400, and 400–850 °C, respectively. Thus, at the first stage, the weight loss accompanied by an endothermic effect (56 °C) amounts to 4.8% and is associated with the removal of the residual solvent of sorbed atmospheric gases. The subsequent increase in temperature leads to a more intense reduction in the sample mass (Δm = 21.2%), which is explained by the semi-product decomposition with the formation of NiCo_2_O_4_ oxide. In this case, energy absorption also occurs, and the minimum of the corresponding endo-effect is around 274 °C. The next temperature increase is associated with a significantly less intense decrease in mass, which noticeably accelerates around 750 °C due to the decomposition of the formed NiCo_2_O_4_ oxide and oxygen release, which agrees well with the presence of the corresponding endothermic effect on the DSC curve at 838 °C. Thus, the weight loss in the temperature range of 400–850 °C was about 5.6%, and the value of the total weight loss over the whole investigated temperature range was 31.7%. Thus, according to the synchronous thermal analysis results, the optimal mode of semi-product additional heat treatment (400 °C, 5 h), which provides complete material conversion and preservation of its highly dispersed state, was selected.

**Figure 3:**
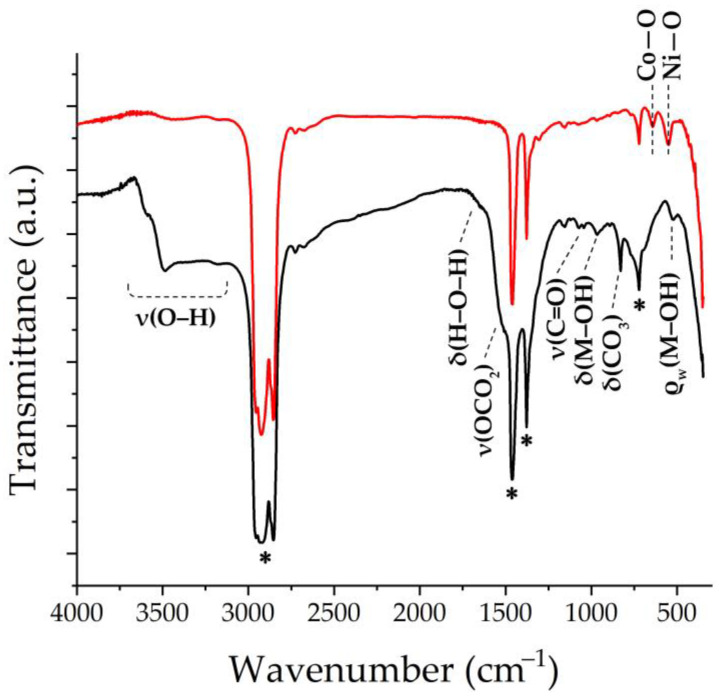
FTIR spectra of the obtained semi-product (black) and NiCo_2_O_4_ oxide (red) (marker “*” indicates Vaseline oil absorption bands).

The set of functional groups in the resulting semi-product and oxide nanopowder was investigated using FTIR spectroscopy (Figure 3). Thus, in the semi-product spectrum, the absorption band around 1530 cm^−1^, partially overlapping with the signal from the Vaseline oil, refers to the ν(OCO_2_) stretching vibration. The bands around 1060 and 830 cm^−1^ are related to ν(C=O) and δ(CO_3_), respectively, and the bands with maxima at 965 and 522 cm^−1^ are probably related to δ(M–OH) and ρ_w_(Co–OH) vibrations, respectively. This set of absorption bands is characteristic of carbonate hydroxide hydrate [57]. In the region of 3100–3700 cm^−1^, a medium-intensity band related to the ν(O–H) stretching vibration is observed [58]. In the spectrum of the oxide powder resulting from the additional heat treatment of the semi-product, there are two absorption bands with maximums at 641 and 550 cm^−1^ related to the (Co-O) and (Ni-O) group vibrations, respectively, which are characteristic of NiCo_2_O_4_ with a spinel structure [59].

**Figure 4:**
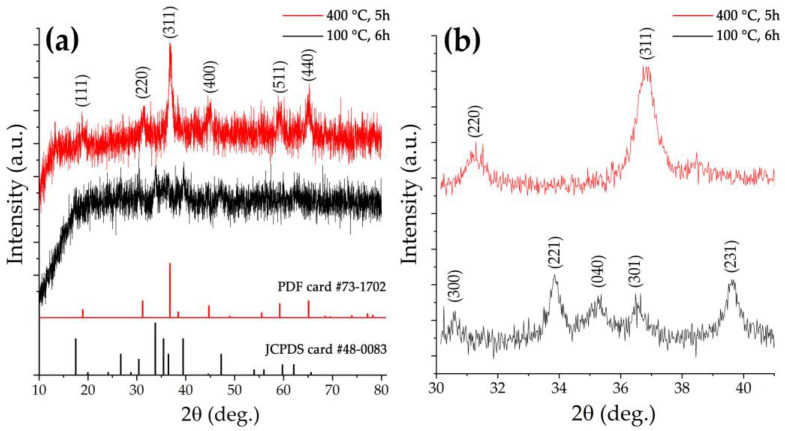
XRD patterns of the semi-product (black) and the oxide powder (red) obtained by its additional heat treatment ((**a**) survey, (**b**) with increased signal accumulation time in the 2θ interval 30–41°).

The crystalline structure of the obtained semi-product and oxide powder was studied by X-ray diffraction analysis (Figure 4). The view of the X-ray diffraction pattern of the semi-product shows a rather low crystallinity degree of the material. At the same time, a 2θ range 30–41° XRD pattern, obtained with extended signal accumulation time, shows a set of reflections characteristic for nickel-cobalt carbonate hydroxide hydrate of composition [57] M(CO_3_)_0.5_(OH)·0.11H_2_O (where M is Ni^2+^ and Co^2+^) (JCPDS card #48-0083) quite well. Additional heat treatment of the powder at 400 °C, as seen from the corresponding XRD patterns, leads to the complete decomposition of the semi-product with target oxide formation of a spinel structure (space group Fd3m, PDF # 73-1702). The full-profile analysis of the obtained NiCo_2_O_4_ powder XRD pattern made it possible to estimate the average size of the coherent scattering region (CSR), the value of which was 12 ± 2 nm. No crystal impurities were found in the oxide powder structure. Thus, the chosen regime of a semi-product additional heat treatment proved to be optimal, providing complete semi-product conversion and maintaining high dispersity of the formed NiCo_2_O_4_ oxide powder without its partial decomposition, which may occur in the case of exceeding a certain temperature.

**Figure 5:**
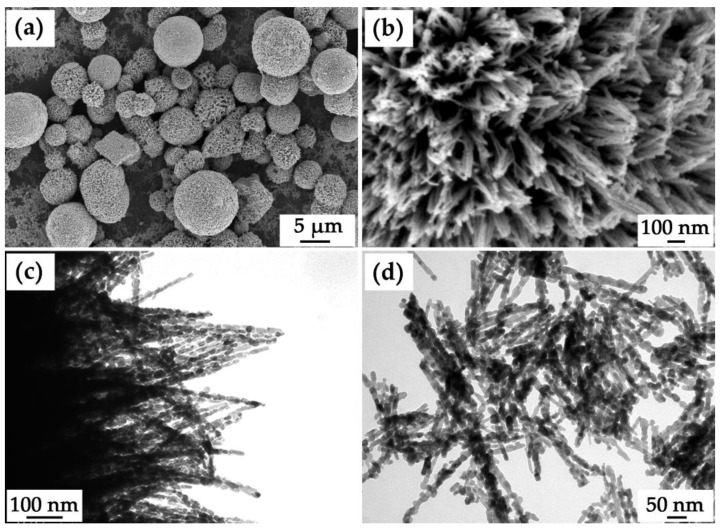
Microstructure of the obtained NiCo_2_O_4_ powder ((**a**,**b**) SEM; (**c**,**d**) TEM).

The microstructural features of the prepared NiCo_2_O_4_ powder were examined by scanning electron microscopy (Figure 5a,b) and transmission electron microscopy (Figure 5c,d). Thus, according to the scanning electron microscopy data, the main powder fraction consists of hierarchically organized microspheres with diameters of 3–10 μm organized from nanorods about 20 nm thick that spread out from their centers. As can be seen from the micrograph (Figure 5b), nanorods are connected at their ends on the outer surface of the spherical formations, forming bundles of 5–10 one-dimensional structures. It should be noted that individual nanorods whose geometrical parameters are close to the structural elements making up the microspheres are also observed as a second fraction within the powder. By means of transmission electron microscopy, the surface of NiCo_2_O_4_ microspheres (Figure 5c) and the structure of individual nanorods (Figure 5d) were studied in more detail. In particular, the individual nanorods and one-dimensional nanostructures within the microspheres were also found to be composed of smaller particles. Thus, the corresponding primary particles have a slightly elongated shape, their length is about 20–30 nm, and their thickness is about 10 nm, which agrees well with the average CSR value calculated from the XRD data. Thus, the obtained oxide powder has a developed microstructure and several levels of organization, which is preferable when selecting components for supercapacitor electrodes.

**Figure 6:**
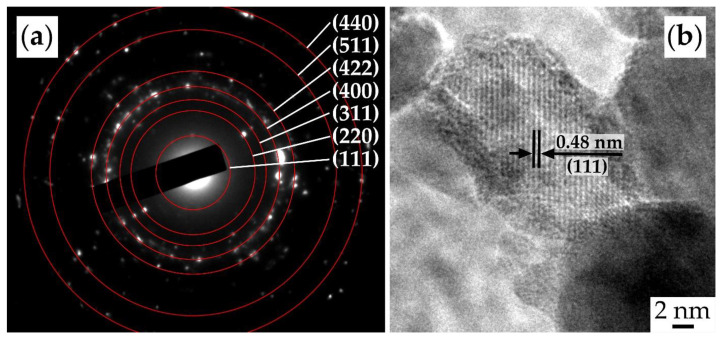
SAED (**a**) and HR-TEM results (**b**) for the obtained NiCo_2_O_4_ powder.

The oxide powder used to form the NiCo_2_O_4_–CP electrode was also studied using SAED (Figure 6a). The results indicate that the synthesis yielded well-crystallized particles with a spinel-like structure (space group Fd3m, PDF # 73-1702) characterized by corresponding crystallographic planes. The HR-TEM image for a single NiCo_2_O_4_ nanoparticle (Figure 6b) allows us to observe lattice fringes corresponding to the crystallographic plane (111) with an average interplanar distance of 0.48 nm.

### 3.2. Characterization of the Printed NiCo_2_O_4_ Film

**Figure 7:**
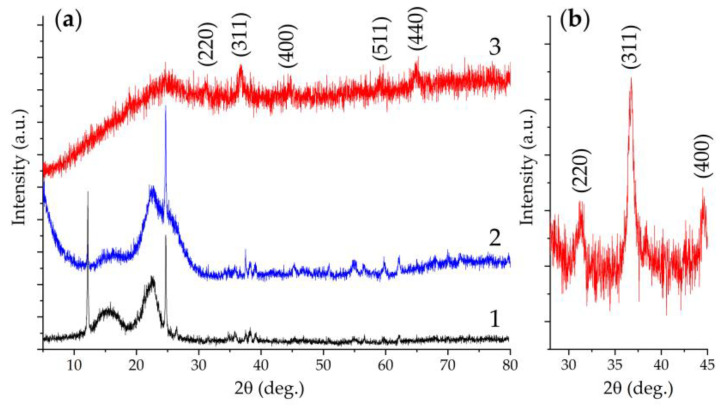
Survey diffractograms (**a**) of the paper support (1), the CP substrate located on it (2), and NiCo_2_O_4_–CP electrode (3), as well as the NiCo_2_O_4_–CP electrode XRD pattern at 28–45° 2θ range with increased signal accumulation time (**b**).

The crystalline structure of the original CP substrate and after applying the NiCo_2_O_4_ film to its surface was studied by XRD analysis (Figure 7). From the original substrate XRD pattern (Figure 7a, 2), it is clear that besides the halo characteristic of such materials in the region of 20–30°, there are also sufficiently intense narrow reflexes related to the paper support (Figure 7a, 1), on which the sample was placed during the analysis. This fact indicates a rather porous structure of the used CP substrate. The survey X-ray diffraction pattern for the NiCo_2_O_4_–CP electrode contains a set of reflections characteristic of the cubic crystal structure of the NiCo_2_O_4_ oxide (space group Fd3m, PDF # 73-1702). To better identify the oxide particle structure in the 2θ range of 28–45°, a diffractogram was recorded with an extended to 2.0 s signal accumulation time (Figure 7b). The results obtained suggest that when the active component was deposited on the CP substrate surface, its crystal structure was preserved, and no crystal impurities were detected.

**Figure 8:**
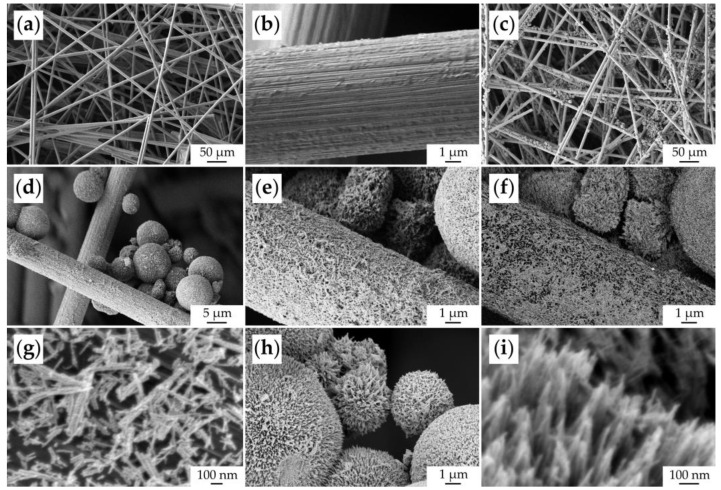
Microstructure of the original CP substrate (**a**,**b**) and NiCo_2_O_4_–CP electrode (**c**–**i**) (based on SEM data).

The microstructure of the original CP substrate and the fabricated NiCo_2_O_4_–CP electrode was studied by scanning electron microscopy (Figure 8). Using the SE2 detector, it was shown that the original substrate was porous and consisted of carbon fibers about 7 μm thick (Figure 8a,b). In the case of the NiCo_2_O_4_–CP electrode, one can observe that the NiCo_2_O_4_ spherical hierarchically organized structures with a diameter of 5–10 μm are quite uniformly fixed on the surface of the carbon fibers and in the pores between them (Figure 8c,d) upon functional ink application. A film of oxide nanorods about 100 nm thick was formed on the surface of the carbon fibers by self-organization (Figure 8e), which is clearly visible in phase contrast mode with an ESB detector (Figure 8f) or In-lens secondary electron detector (Figure 8g). It can also be observed that these nanorods are composed of smaller particles (about 10 nm in size). A more detailed microstructure study of the oxide microspheres also showed that they consist of similar nanorods spreading out from the spherical structure center and gathering at their ends into bundles (Figure 8h,i). Thus, when applying the NiCo_2_O_4_ film, the inheritance of the microstructural features of the corresponding oxide nanopowder obtained by the hydrothermal method is observed. At the same time, due to the printing of the active component film, the CP substrate surface has become much more developed, which is necessary to ensure the supercapacitor electrode performance.

**Figure 9:**
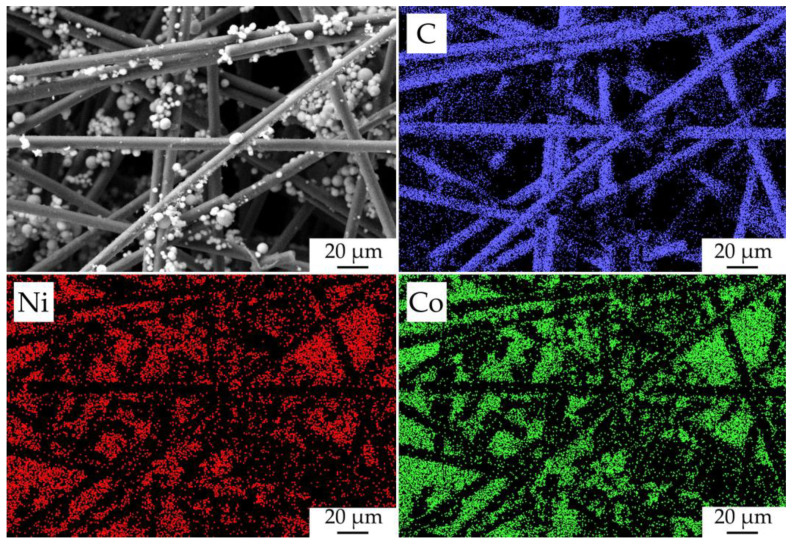
Microstructure of the fabricated NiCo_2_O_4_–CP electrode and the corresponding maps of chemical element (C, Ni, and Co) distribution on its surface.

The character of the fabricated NiCo_2_O_4_–CP electrode surface filled with oxide nanoparticles was studied by mapping the corresponding chemical element distribution on its surface (Figure 9). As a result, the elemental maps of nickel and cobalt showed that the hierarchically organized NiCo_2_O_4_ nanostructures are sufficiently evenly and efficiently distributed in the pore space of the CP substrate. Due to the fact that a relatively thin film of oxide nanorods was formed on the carbon-fiber surface, the signal from it is very weak and virtually invisible on the nickel and cobalt distribution maps. When analyzing the studied material surface, the target metal ratio in the functional oxide layer composition (n(Co)/n(Ni) = 2.07), as well as the absence of any impurities, was also confirmed.

**Figure 10:**
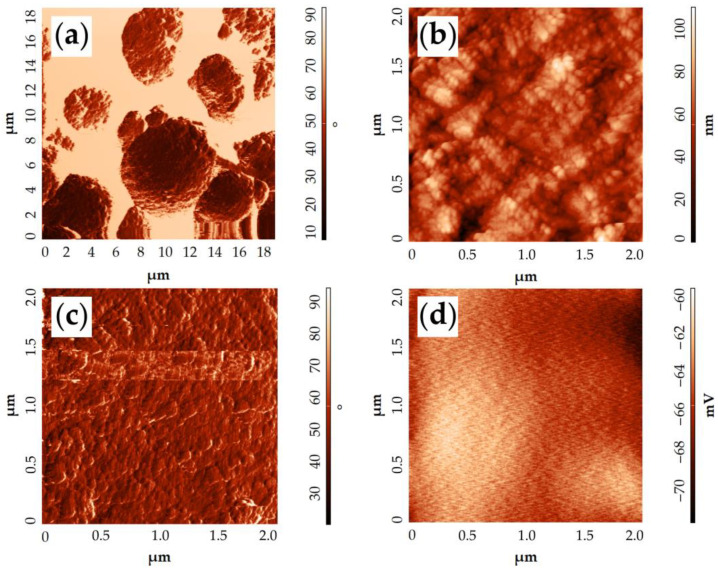
AFM results of the NiCo_2_O_4_–CP electrode surface examination: topography in the phase contrast mode (**a**,**c**), topography (**b**), surface potential distribution map according to KPFM data (**d**).

The obtained NiCo_2_O_4_–CP electrode surface was also examined by atomic force microscopy (Figure 10). From the corresponding phase contrast (Figure 10a,c) and topography images (Figure 10b), it is clear that the NiCo_2_O_4_ particles have a slightly elongated shape 30–40 nm long and about 15 nm wide while being organized into large microspheres that vary in diameter from 4 to 6 μm. The surface potential distribution map derived from KPFM data indirectly indicates a sufficiently high conductivity of the resulting material. This conclusion can be made on the basis of a very low dispersion (12 mV) between the values of the surface potential in some points of the scanned sections. This is also evidenced by the appearance of the map, where there are no clearly distinguishable areas of increased or decreased potential. The electronic work function of the material surface was determined using the KPFM results and amounted to 5.08 eV. Such a value for the NiCo_2_O_4_ oxide is somewhat lower than those found in the literature and obtained by analogous methods [60,61]. This may be related to the peculiarities of the material synthesis technique used in our study, resulting in a special material microstructure and affecting the material composition. For example, Pathak et al. [62] have shown in their work that when the number of oxygen vacancies in NiCo_2_O_4_ increases, the work function value decreases. Consequently, it is likely that in our case the material with a slightly higher content of oxygen vacancies is formed.

Thus, AFM results verify the data on sample microstructures available from SEM, namely that elongated nanoparticles of NiCo_2_O_4_ constitute hierarchic spheres. KPFM results suggest that NiCo_2_O_4_ should exhibit relatively high electrical conductivity and possess a heightened number of oxygen vacancies.

### 3.3. Electrochemical Evaluation

**Figure 11:**
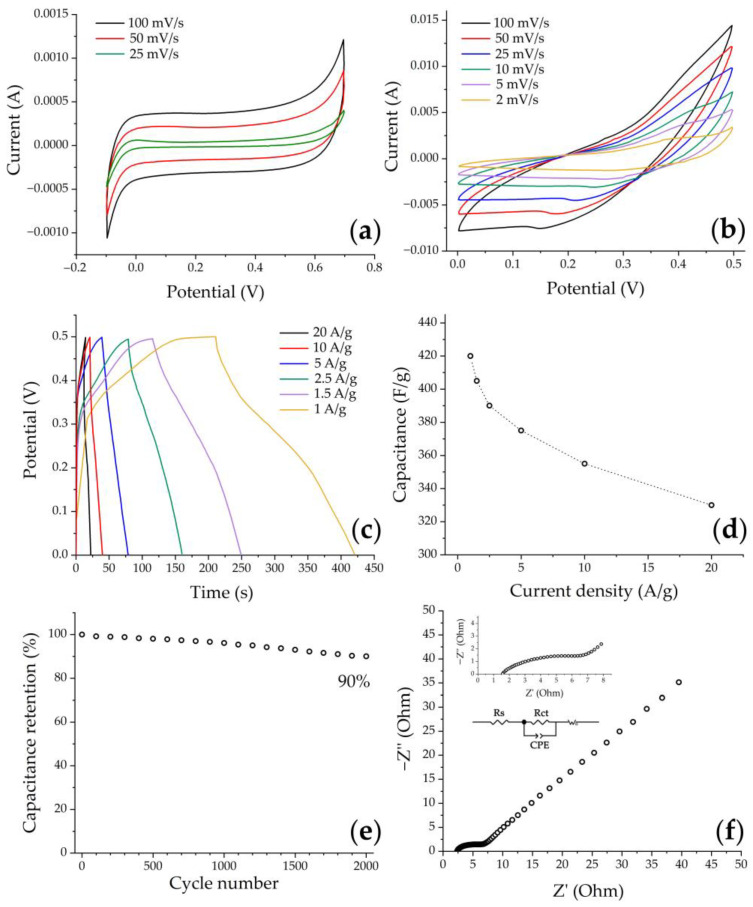
Cyclic voltammogram of clean carbon paper (**a**) and NiCo_2_O_4_–CP electrode (**b**) in 3 M KOH solution obtained at different scan rates, GCD curves at different current densities (**c**), rate capability (**d**), cycling life test at 10 A/g for 2000 cycles (**e**), and the EIS spectrum for the electrode material under study (inset: enlargement of high-frequency part and equivalent circuit diagram) (**f**).

In the first stage of electrochemical studies, the electrochemical behavior of the original carbon paper in a KOH medium (3 mol/L) was studied by cyclic voltammetry. Figure 11a shows typical voltammetry curves of a clean substrate at different scan rates in the potential range from −0.1 to 0.7 V, which correspond to the capacitance due to the electrical double layer effect. It can be seen that the current values on the curves as well as the CV-integrated area in the case of a pure substrate are an order of magnitude smaller compared to the substrate with printed NiCo_2_O_4_ film (Figure 11b). This indicates the insignificant contribution of carbon paper to the NiCo_2_O_4_–CP electrode functional characteristics, and that the hierarchically organized NiCo_2_O_4_ active layer provides the main part of the electrode accumulated capacitance. On the CVA curves corresponding to the active electrode layer under study, a pair of redox peaks characteristic of this material can be observed in the 0–0.5 V range, indicating the presence of a pseudocapacitance contribution to the total capacitance due to the following processes [12,63,64]:

NiCo_2_O_4_ + OH^−^ ↔ NiOOH +2CoOOH + e^−^ (2)

NiOOH + e^−^ ↔ NiO + OH^−^ (3)

CoOOH + OH^−^ ↔ CoO_2_ + H_2_O + e^−^ (4)

CoOOH + e^−^ ↔ CoO + OH^−^ + e^−^ (5)

The peak currents increase with the increasing scanning rates, suggesting that this material is beneficial for fast redox reactions. Moreover, when increasing the scanning rates from 2 to 100 mV/s, one can observe that the CVA curves (Figure 11b) show a slight shift of the anode peaks (oxidation processes) towards the positive region, while the cathode peaks (reduction processes) shift towards the negative region. Such a phenomenon is associated with ohmic resistance and polarization processes occurring at the electrode/electrolyte interfaces, which in turn are caused by the kinetics of electron and electrolyte ion transfer processes [65]. However, the shape of the curves (Figure 11b) does not undergo significant changes, which may suggest a sufficiently high stability and reversibility of the electrode.

In order to evaluate the specific capacitance as well as cyclic stability of the NiCo_2_O_4_–CP electrode, a galvanostatic charge–discharge method was used within the same three-electrode scheme in the 0–0.5 V voltage window while varying the current density from 1 to 20 A/g. All curves (Figure 11c) show the general tendency of nonlinear voltage change with increasing time during the charge–discharge cycle, which confirms the occurrence of Faraday processes when cycling the material. The specific capacity values of the NiCo_2_O_4_–CP electrode were calculated using equation (2) and GDC test data. The capacitance was found to decrease from 420 to 330 F/g when the current density was increased from 1 to 20 A/g (Figure 11d). So, in this case, the capacitance decrease was 22%, which indicates a fairly good rate capability of the obtained electrode. The observed dependence of the capacitance value on the current density is typical for such materials and is related to the fact that the charge transfer process at higher current values is complicated by the limited diffusion time of electrolyte ions into the electrode material. A cycling life test for 2000 cycles at 10 A/g (Figure 11e) showed that the specific capacitance value was retained by 90%, which may be due to the nanoarchitecture features of the electrode active layer. For example, in papers [63,66], it was previously shown that electrode materials based on nickel-cobalt spinel with a nanorod structure exhibit increased capacitance values as compared to their analogs consisting of nanoplates and microspheres since they enable stabilization of the material volume change during incorporation/extraction of hydroxide ions into it according to schematic reactions 3–5. At the same time, combining such nanorods into spherical agglomerates, as shown in the studies [41,67], can give an additional increase in the capacitance due to a three-dimensional mesoporous network formation, providing an increased contact area between electrolyte ions and the electrode material surface, as well as ensuring additional pathways of space charge transfer.

The use of electrochemical impedance spectroscopy allowed us to further study the functional characteristics of the obtained electrode material. The impedance spectrum presented in Figure 11f, recorded in the 100 kHz–0.1 Hz frequency range, consists of a semicircle (high-frequency range) and a linear component (low-frequency range). The intersection area of the impedance hodograph with the real coordinate axis in the high-frequency range corresponds to the ohmic resistance of the system under study (R_s_), including the total electrode resistance and the electrolyte ion resistance. The diameter of the semicircle in the high-frequency range corresponds to the charge transfer resistance (R_ct_) [66]. In our case, the investigated NiCo_2_O_4_–CP electrode demonstrates relatively low values of Rs (1.52 Ohm) and Rct (7.51 Ohm), which may indicate rather active ion transport at the electrode–electrolyte boundary, and also conditions the high rate performance of the electrode [68]. The linear component of the obtained impedance spectrum can be described by the Warburg impedance, corresponding to the electrolyte ion diffusion to the electrode material active centers.

**Figure 12:**
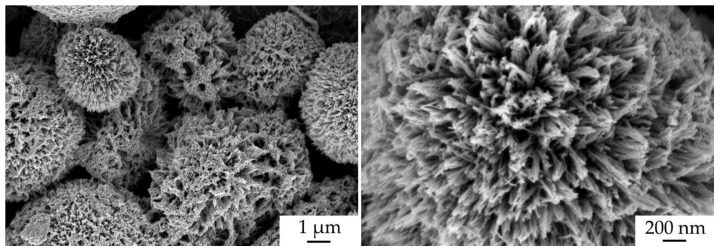
NiCo_2_O_4_–CP electrode microstructure after electrochemical measurements (according to SEM data).

The surface microstructure of the NiCo_2_O_4_–CP electrode was also studied after the electrochemical measurements (Figure 12). As can be seen from the corresponding micrographs, the character of the NiCo_2_O_4_ film morphology was preserved as well as at the stage of active material layer printing, and the anisotropic one-dimensional oxide particles are hierarchically organized into spherical formations. No fundamental changes manifested in the transformation of the shape and size of the particles are observed in this case.

Thus, the capacitance values of the NiCo_2_O_4_ film printed on the carbon paper surface correspond to and in some cases exceed the values presented in the works on developing supercapacitor electrodes based on transition metal oxides (Table 1). It can be seen that the synergistic effect of the presence of two or more transition metals in the material composition leads to an increase in the specific capacitance of the target electrode. At the same time, the electrode formed in our work demonstrated high cyclic stability, which is an important factor in the design of reliable devices with a long service life. A further increase in the specific capacitance of the proposed electrode material can be achieved by additional optimization of the functional ink formation technique (for example, by introducing binders, more conductive additives, or increasing the mass fraction of the solid phase), as well as the printing process parameters. However, it was demonstrated that the proposed synthesis and printing technology provides an opportunity for the efficient automated formation of the corresponding miniature electrode nanostructures as promising components for flexible planar supercapacitors.

## 4. Conclusions

The hydrothermal synthesis of a nanosized NiCo_2_O_4_ oxide with several levels of hierarchical self-organization was studied. By means of XRD analysis and FTIR spectroscopy, it was established that under the proposed synthesis conditions a nickel-cobalt carbonate hydroxide hydrate (M(CO_3_)_0.5_(OH)·0.11H_2_O, where M is Ni^2+^ and Co^2+^) is formed as a semi-product, which, according to synchronous thermal analysis, completely decomposes with the target oxide formation of a spinel structure during the additional heat treatment at 400 °C for 5 h. Based on the scanning electron microscopy data, the main powder fraction consists of hierarchically organized microspheres 3–10 μm in diameter organized from nanorods about 20 nm thick, which spread out from their centers. The second fraction in the powder composition also includes individual nanorods whose geometrical parameters are close to the structural elements of which the microspheres are composed. Using transmission electron microscopy, it was determined that single nanorods and one-dimensional nanostructures within microspheres also consist of smaller particles with a slightly elongated shape, and their length is about 20–30 nm and thickness is about 10 nm, which agrees well with the value of the average CSR size calculated from X-ray diffraction analysis data (12 ± 2 nm). The NiCo_2_O_4_ film was deposited on the carbon paper surface by microplotter printing using functional inks based on the obtained oxide powder. According to the XRD, scanning electron microscopy, and atomic force microscopy data of the obtained NiCo_2_O_4_–CP electrode, it was demonstrated that the crystal structure and microstructural features of the oxide particles are preserved when applied to the flexible substrate surface. The electrochemical characteristics of the obtained sample were studied; in particular, the specific capacitance values were calculated, which increased from 330 to 420 F/g when the current density decreased from 20 to 1 A/g. A cycling life test for 2000 cycles at 10 A/g showed that the value of specific capacity was retained at 90%, which may be due to the features of the nanoarchitecture of the active layer of the electrode and indicates a sufficiently high stability of the material. Thus, it was demonstrated that the suggested synthesis and printing technology provides an opportunity for the effective automated formation of the corresponding miniature electrode nanostructures as promising components for flexible planar supercapacitors.

## Figures and Tables

**Table 1:** Comparison of the specific capacitance values for electrode materials based on transition metal oxides.

Electrode Material	Technology	Specific Capacitance	Reference
NiCo_2_O_4_ nanosheets	Microwave-assisted heating method	292.5 F/g at 1 A/g	[69]
NiO	Hydrothermal synthesis	132 F/g at 5 mV/s	[70]
Feather-like Co_3_O_4_	Electrodeposition	397 F/g at 20 mV/s	[71]
(Co–Ni–Cu) mixed oxides nanospheres	Galvanostatic electrodeposition	525 F/g at 1 mA	[72]
NiMoO_4_ nanorods	Coprecipitation	255 F/g at 2 mA/cm^2^	[73]
urchin-like NiCo_2_O_4_	Hydrothermal synthesis	420 F/g at 1 A/g	This work

## Data Availability

Not applicable.
